# Ethical Challenges With Smartwatch-Based Screening for Atrial Fibrillation: Putting Users at Risk for Marketing Purposes?

**DOI:** 10.3389/fcvm.2020.615927

**Published:** 2021-01-15

**Authors:** Christopher Predel, Florian Steger

**Affiliations:** Institute of the History, Philosophy and Ethics of Medicine, Ulm University, Ulm, Germany

**Keywords:** ethics, wearable, smartwatch, artificial intelligence, atrial fibrillation, autonomy, justice

## Abstract

**Background:** Atrial fibrillation is the most common persistent arrhythmia. It is associated with increased mortality and morbidity such as stroke. The early detection of atrial fibrillation can significantly reduce the risk of stroke through preventive anticoagulation. Smartwatches offer the opportunity to screen for atrial fibrillation in the general population. This paper aims to analyze the ethical challenges associated with screening for atrial fibrillation using smartwatches.

**Methods:** This is an ethical analysis. The methodology is based on the principle-orientated approach of Beauchamp and Childress. The principles of beneficence, non-maleficence, justice, and autonomy have to be guaranteed given the influence of private companies, privacy protection, liability and doctor-patient-relationship. The work is based on a systematic literature research.

**Results:** There is currently no evidence that screening for atrial fibrillation with smartwatches improves the outcome and reduces the number of adverse events. The high number of false-positive results can lead to harm. The principle of non-maleficence is violated. The over-reliance on and the lack of adequate education by smartwatches can worsen the doctor-patient relationship. However, the relationship can also be improved by the proactive participation of the patient, which leads to greater autonomy, compliance and in the end beneficence. Since smartwatches are consumer goods, there is a risk for greater disparities in the poor and rich population. There is also a risk of discrimination against ethnic minorities due to underrepresentation in training data and study cohorts. The principle of justice is violated. The storage of sensitive medical data by private companies also raises many ethical and legal concerns.

**Conclusion:** This analysis has shown that the use of smartwatches to detect atrial fibrillation is currently in an ethical perspective problematic. The lack of evidence and the high number of false-positive results can lead to harm. As smartwatches provide only little information about the possible consequences, informed consent cannot be assumed. Ethical implementation could be archived if doctors provide smartwatches to patients who have been shown to benefit from them. The implementation and education should be managed by the doctor.

## Introduction

Atrial fibrillation is the most common persistent arrhythmia that affects 3% of people over 20 years of age, with greater prevalence in older persons (1). Atrial fibrillation is associated with increased mortality and morbidity, such as stroke or heart failure. Twenty–Thirty percentage of the strokes are caused by atrial fibrillation (2). The early detection of atrial fibrillation can significantly reduce the risk of stroke through preventive anticoagulation therapy (3). Undetected silent atrial fibrillation is common due to many people with missing symptoms (4). The diagnosis is often made after the first thromboembolic event (5). Because atrial fibrillation has a significant impact on the quality of life and is a major cause of health care expenditure, the introduction of screening programs has been considered (6). A study by Lowres et al. shows that a chronic form of atrial fibrillation was found in the course of screening programs in 2.3% of the in an average 64-year-old screening population. Previously undiagnosed atrial fibrillation was found in 1.4% of those over 65 years of age, while the majority of this group would benefit from anticoagulation to prevent stroke (7). Since paroxysmal atrial fibrillation is often overlooked by single measurements, there are concepts for long-term ECG monitoring that increase the sensitivity for the detection of short atrial fibrillation episodes (4). Holter-ECG and invasive heart monitoring significantly increase the detection rate for atrial fibrillation. Due to inconvenience, invasiveness or cost inefficiency, these screening methods are not applicable to everyone (8). Innovations in hardware and software development have opened new opportunities for cost-effective and convenient screening methods. A promising example is the screening for atrial fibrillation with smartwatches. These wearable devices could provide long-term, non-invasive, cost-effective and convenient ECG monitoring. The smartwatches are equipped with a photoplethysmography sensor that can record a tachogram with a built-in camera and an electrical sensor that can record a rhythm strip equivalent to a single lead ECG. The signals of the tachogram and the ECG-lead are analyzed and evaluated by an intelligent algorithm that can detect irregular heart rhythms, such as atrial fibrillation with high accuracy. The algorithm, based on photoplethysmography, detects irregular heart rhythms and notifies the users who have activated the function. To measure the electrocardiogram, the user must place his finger on the crown of the watch. The user is informed about the interpretation and the non-diagnostic nature of the results before the measurement. The algorithm has a sensitivity of >98% and a specificity of >99% to classify the results as sinus rhythm, atrial fibrillation, or not conclusive (9). In 2019 Perez et al. carried out the Apple heart study. Over 400.000 study participants were screened for atrial fibrillation using the Apple watch. The study showed that 84% of the notifications that the users received matched atrial fibrillation. Fifty seven percentage of the notified patients contacted healthcare providers outside of the study (10). Apple's algorithm for the detection of atrial fibrillation was the first one to be approved for the consumer market by the US Food and Drug Administration (11). Atrial fibrillation screening using smartwatches has not been included in the clinical guidelines but could become a reality soon.

Screening for atrial fibrillation with smartwatches offers the possibility of preventive treatment and could lead to an improved quality of life for many people. But it also raises ethical questions that will be discussed in this paper. One question is whether the number of false positive results violates the principle of non-maleficence. The unequal access to healthcare and the participation of different user groups such as ethnic minorities and the elderly are also discussed.

## Methods

This work is an ethical analysis. The method is based on the principle-orientated approach of Beauchamp and Childress. This approach is widely used in clinical practice and represents an important standard in medical ethics (12). This work analyzes the principles that are affected by using smartwatches for screening for atrial fibrillation. Due to the special position of wearables and artificial intelligence, the influence of private companies, privacy protection, liability and doctor-patient-relationship were also analyzed.

The work is based on a systematic literature search. PubMed, Google Scholar, Google, BELIT and ScienceDirect were used. First, the research question was defined. A systematic literature search was then carried out using the keywords “smartwatch,” “wearable,” “atrial fibrillation,” “screening” and “ethics” in various combinations. The results were selected for relevant articles. As algorithms to detect atrial fibrillation have been introduced in recent years, emphasis has been placed on the latest literature. Studies were selected that analyze the detection of atrial fibrillation using smartwatches. Studies that analyze the detection of atrial fibrillation by doctors or non-smartwatch-based algorithms were excluded. The results were then summarized, structured and discussed in a narrative synthesis. After removing duplicates and screening the title and abstract, a detailed assessment of 65 works was carried out. Of the 65 papers, 22 did not meet the inclusion criteria described above, so that 43 papers were included in this ethical analysis to answer the research question.

## Results

### Non-maleficence

Screening for atrial fibrillation with smartwatches hypothesizes that early detection would prevent adverse events, such as stroke, by treating with anticoagulants (13). As with any screening program, screening for atrial fibrillation will harm a part of the screening population. False-positive notifications create stress and anxiety for the misdiagnosed person. Follow-up diagnostics, for example with implantable devices and possible treatment with anticoagulants, will decrease the quality of life and can lead to dangerous events, such as iatrogenic bleeding. Although the specificity of the algorithm used by the Apple smartwatch is very high, many people have still been notified of arrhythmia without having atrial fibrillation (10). It is also important to note that the prevalence of atrial fibrillation is higher in the older population. In the USA only 4,6% of smartwatch users are older than 65 years (14). This results in a higher number needed to screen and a smaller positive predictive value with a higher potential for unnecessary diagnostics and treatment (15). Studies show that nearly 1 million per 10 million people screened would get a false-positive result (16). While unselected screening appears to be ineffective in the general population, systematic opportunistic screening for patients with a high probability of atrial fibrillation could be a cost-effective use of resources (17). Manish et al. note that the relationship between atrial fibrillation and stroke is not well-enough understood to screen and treat for it. There is still little evidence whether patients with subclinical atrial fibrillation should receive anticoagulation (18). The U.S. Preventive Services Task Force stated that the current evidence is inadequate to determine the potential harm of unnecessary follow-up diagnostic and treatment caused by ECG screening (19). The principle of non-maleficence is violated regarding screening.

### Beneficence

The use of the ECG function of the smartwatch could be beneficial for patients with diagnosed atrial fibrillation. Patients who have been treated with antiarrhythmic therapy with the “pill in the pocket” method could benefit from immediate rhythm detection. It could also be useful for assessing the effectiveness of interventions such as catheter ablation. This offers the opportunity of personalized care and improved quality of life (20).

### Patient Autonomy

#### Increased Empowerment of the Patient

The detection of atrial fibrillation with smartwatches could offer the opportunity for increased participation of the user in his health management. By actively using the ECG-feature, the user takes a proactive role in preventive self-care. Patients who have already been diagnosed with atrial fibrillation could proactively engage in the surveillance of their current therapy and find the best therapy with their doctors (21). Smartwatches give patients more and easier access to their health data compared to normal home monitoring devices, such as Holter-ECG or implantable devices. If the patient has a smartwatch and can operate it, stigma could be reduced compared to visible monitoring devices. In addition, they offer flexibility and comfort for the patient and thus increase acceptance for long term home monitoring (22). This could lead to an informed patient gaining a deeper understanding of his illness and thereby gaining greater autonomy. PewInternetResearch showed that 69% of US adults track at least one health indicator. Forty six percentage of the participants said that tracking changed their approach to maintaining their health. In people with chronic diseases, such as atrial fibrillation, tracking even had a greater impact (23).

#### Informed Consent

Smartwatches make it possible to screen a large population at low cost for atrial fibrillation without the user knowing that his heart rhythm is being recorded. It is important that the user still has the choice of whether to participate in the screening program or not. Therefore, informed decision-making is required. It is known from other screening programs that decision-making is often not well-informed (24). Users need to be informed of the consequences of false-positive and false-negative results and the diagnostic procedures that may be required for further assessment. In addition, it is important to inform the user about the possibility of incidental findings that could result from the following diagnostics. The Apple Watch algorithm can only classify the heart rhythm as sinus rhythm or atrial fibrillation. However, further findings could show up in the following examination. Ethical guidelines for incidental findings in genetics and imaging have been established. When screening for cardiac arrhythmia, the patient should also be informed of the extent to which incidental findings are being communicated, taking into account the right not to know and the protection of patients who are unable to give consent (25). It is also important to understand what atrial fibrillation is, what the possible dangers are and what could be done about it. For using the ECG-function of the Apple watch the user must consent. Previously he is briefly informed about atrial fibrillation and the ECG-function. An education like this could not replace a personal education by a doctor. It is not possible to assess whether the user has fully understood the risks and consequences of a diagnostic test for atrial fibrillation. In addition, it is not possible to ask questions like in a real visit with a doctor. Current smartwatch applications do not provide the necessary education for informed consent. The patient education must be given by a doctor and requires a personal conversation.

### Doctor-Patient-Relationship

#### Over-reliance on Smartwatches

The use of smartwatches for detecting atrial fibrillation could lead to an empowerment of the patient, but also an impaired doctor-patient-relationship. Patients could overestimate the accuracy and potential of the diagnostic capabilities of smartwatches. The importance of a classification of the results in an overall context by an experienced doctor and the limits of smartwatches are not obvious to everyone (22). The algorithm implemented by Apple can only distinguish atrial fibrillation from sinus rhythm. Other arrhythmia or severe cardiac conditions like heart attack cannot be diagnosed by a smartwatch. People who are not well-educated about the limits of the algorithms may feel in a false sense of security. After the measurement, the Apple Watch informs the user that severe cardiac conditions should be examined by a doctor and that the algorithm can only detect atrial fibrillation. Patients with acute illnesses, elderly people, or those with little medical understanding could misinterpret this warning.

An overuse and over-reliance on technologies like smartwatches can also lead to mental health issues. Improper application and medicalization of everyday life can lead to stress, especially in hypochondriacal individuals. Home monitoring of atrial fibrillation could lead to fewer face-to-face doctor visits. There are big differences between a smartwatch or a doctor telling a patient that he has or does not have atrial fibrillation. A doctor's confirmation to be healthy could bring more relief than a negative ECG-result from a smartwatch. The use of smart wearable devices focuses on the aggregation of biomedical data and does not take a holistic view of the patient (26). The over-reliance on smartwatches and the lower number of face-to-face doctor visits lead to a violation of the principle of non-maleficence. The false sense of security due to the exclusion of atrial fibrillation by the algorithm could lead to a delayed diagnosis of acute diseases.

#### Improvement of the Relationship

If the implementation of smartwatches to detect and monitor atrial fibrillation is led by clinicians and if patients are educated about the opportunity of early detection, treatment of atrial fibrillation, the risk of false-positives and the dangers of anticoagulation, the doctor-patient-relationship could be improved. The patient's participation in the diagnostic and therapeutic process and the increased availability of home aggregated information could improve the communication and trust between the patient and the doctor. Existing applications that support symptom documentation for chronically ill patients show that the patients are well-informed about their illness and that decisions between patients and doctors have been improved (27).

### Justice

#### Increasing Inequalities

To ensure justice, everyone should have equal access to healthcare regardless of race, gender, sexual orientation, religion, socioeconomic status or age (12). Because smartwatches are expensive consumer products, there are socioeconomic and demographic differences in the adaptation to these technologies (20). Smartwatches are mainly bought by young persons with higher socioeconomic status (28). Noting the fact that low income correlates with increased risk of atrial fibrillation, the disparity between rich and poor could become even greater (29). If smartwatches are used as diagnostic tools for screening and are still just consumer goods, access to healthcare will be lower for the low-income population. Health insurers, such as AOK in Germany or Aetna in the USA, offer a bonus for people who can prove good fitness records on their smartwatches. This leads to further discrimination against people who cannot afford smartwatches (30). It is also possible that people are forced to behave in a health-conscious manner and that in future the refusal of screenings with smartwatches is considered irrational (31).

Another problem regarding insurance is that individuals who want to make insurance for disability or health must report their diagnosed illnesses. Atrial fibrillation is usually found in older people with longer fibrillation periods. By detecting very short periods of atrial fibrillation in a young cohort, users who may have never been diagnosed become patients and may have insurance disadvantages (32).

Woman with a low level of education are at higher risk of atrial fibrillation than men (29). Women with atrial fibrillation have an increased risk of death compared to men with atrial fibrillation (2). With only 29% of smartwatch users being female, this could lead to unequal health care between men and women.

High-quality photoplethysmography- or ECG-recordings must be made to analyze the heart rhythm for the detection of atrial fibrillation. In particular, the older population that could benefit most from the detection is often unfamiliar with the use of smartwatches (15). If smartwatches are used for screening programs and the older population is not adequately trained in their use, this can lead to injustice.

The studies that evaluated the accuracy of the algorithm for detecting atrial fibrillation were not representative. The participants of the Apple heart study were on average 41 years old and 68% were white (10). If the algorithms for the detection of atrial fibrillation are not trained and evaluated for a representative group, minorities could not benefit from screening programs due to a lack of algorithmic accuracy and evidence. This could lead to discrimination against ethnic minorities and aggravation of health disparities (33).

#### Increased Justice

The detection of atrial fibrillation with smartwatches also offers the opportunity of increased participation of large population groups in preventive healthcare.

A study carried out in rural India showed that hard-to-reach populations with limited access to healthcare can benefit from the atrial fibrillation detection by smartwatches. Screenings by village health workers could improve rural population access and participation in health care (34).

An objective algorithm that has been well-tested and evaluated for a broad population could provide the same quality of care for everyone. While the standard of care varies between doctors and their prejudices, an algorithm developed and implemented according to ethical principles could offer a consistently high quality of care (35).

### Private Industry

#### New Research Methods

It is important to say that the participation of private companies in health research has opened many new opportunities. Large investments in the development of innovative technologies and extensive studies, such as the Apple heart study, enable new ways of research and healthcare. The Apple heart study showed that it is possible to recruit many study participants and to carry out a study using digital devices like smartphones and smartwatches. Studies like the Apple heart study could provide a better understanding of atrial fibrillation. Smartwatches offer the opportunity to measure a range of vital parameters in the daily life of a broad population. The research community could benefit from the infrastructure and know-how of the private sector (33).

#### Data Protection

However, the use of direct-to-consumer products, such as smartwatches, in healthcare raises many ethical and legal concerns. One problem is that the companies own and store health data from users who are unsure of what data are stored and how they are used (20). Apple mentioned that the algorithm used to detect atrial fibrillation is proprietary. To assess security and data protection, it would be necessary to analyze the data with which the algorithm was trained and the way it processes new data (9). Data protection laws in the EU do not always apply to health data shared in wearable devices. Depending on the terms and conditions, data collected by smartwatches can be shared or used by companies for various purposes (33). Often the user does not read or fully understand which data are stored and how they are used (36). The data collected and analyzed in the Apple heart study were only available to researchers and not to any Apple employees. But even if data is encrypted, it is possible to reidentify anonymized data (37).

Most people are more willing to share health-related data with public research institutes than private companies (38). In the Apple heart study, Apple collaborated with Stanford University. The collaboration with trustworthy public research institutes could tempt users to share more sensitive data with private companies. Many complex legal questions remain unanswered when it comes to data protection.

#### Privatization of Research

Furthermore, it is problematic that the data collected is not passed on and made available to the public scientific community. If private companies own the infrastructure and the data sets needed for further research, public research institutes become dependent (33). Research in the field of artificial intelligence is becoming increasingly dependent on the commitment of private companies. Since there are only a few companies that have the know-how and the resources to implement diagnostic tools with artificial intelligence, monopolies arise (39). A monopoly of a few companies could lead to a violation of the medical profession's autonomy. The treating doctor is no longer fully involved in the decision-making process. The German Ethics Council sees the monopoly of large software companies as an inhibition of chances and justice of participation. The developments of the companies are largely intransparent, which makes it difficult for third parties to critically reflect on the methods and data used. At the same time, the company's strong market positions leads to a loss of plurality (40).

#### Bypass of Expert Consensus

By introducing diagnostic tools, such as the detection of atrial fibrillation, the private sector has bypassed expert consensus to introduce screening in the general population. Traditionally the scientific community conducts research, discussion and evaluation before screenings or diagnostics are made available to the general public (41). The alteration of this process by the private sector creates various problems. There is currently no evidence that screening for atrial fibrillation in a young group improves the prevalence of stroke and the outcome.

The diagnostic for screening is no longer selected and provided by the doctor. Much more, companies develop and provide the diagnostic tools for the patient. The ethics, prejudice and logic that influence decisions between a doctor and a patient are affected by private companies (40). The autonomy of the physician could be reduced, because the decision whether a person should be screened for a disease is not made by a scientific society or a doctor, but by private companies. This unfiltered screening could put the doctor in a difficult situation: The diagnosis of young people who have only asymptomatic short episodes of atrial fibrillation leads to a dilemma. The doctor must decide whether to treat the patient with anticoagulants and risk complications, such as iatrogenic bleeding and reduced quality of life, or whether not to treat the patient with an increased risk of stroke. There is currently little evidence to answer this question, so the doctor must make a decision that always has a disadvantage. Only the doctor is responsible for the consequences of this decision. The companies that have led to this conflict do not take any responsibilities and often do not inform patients about the problems that could arise.

## Discussion

This analysis has shown the ethical problems that arise with the detection of atrial fibrillation with smartwatches. Four ethical issues have proven to be particularly critical: Evidence-based benefit of screening for atrial fibrillation, the increasing inequalities in healthcare, influence of the private industry and data protection.

The basis for ethical implementation of smartwatches for the detection of atrial fibrillation is the proof of a benefit that exceeds the harm for the user. There are currently no studies that could prove a benefit from screening for atrial fibrillation in the general population. On the contrary, there is a risk of damage to the user from false-positive results. The number of overdiagnosed patients is high due to a large number of young smartwatch users (42). This violates the basic ethical principle of non-maleficence. Screening programs for which there is no evidence-based benefit are usually not introduced. By bypassing common scientific standards and by providing a diagnostic tool to detect atrial fibrillation, private companies put users at medical risk (41). A diagnostic tool should not be implemented in a smartwatch for marketing purposes to maximize profit. The use of artificial intelligence enables cost-effective screening for atrial fibrillation in the general population (17). Despite this possibility, evidence-based research should guide the implementation of screening programs and not vice versa. Regardless of cost and availability, atrial fibrillation screening should only be implemented, if a medical benefit is proven. Evidence must be provided for different population groups in order to meet the criteria for justice. It is important that evidence of a medical benefit is confirmed by independent institutions. Studies conducted by software companies such as the Apple Heart Study should be critically analyzed because of a conflict of interest. The introduction of such algorithms and diagnostic tools must be regulated more strictly by state institutions, such as the FDA and should not be equated with fitness apps. Randomized clinical trials are required before adoption (13). In addition to the Apple Watch, there are other mobile devices that can detect atrial fibrillation in an outpatient setting. One example is KardiaMobile 6L, which is a handheld ECG device that can record a 6-channel ECG in conjunction with an IPhone and detect atrial fibrillation. KardiaMobile 6L has been approved by the FDA for the detection of atrial fibrillation. It has also been approved for individual use without health care professional supervision. The main points of this analysis also apply to devices like the KardiaMobile 6L (43).

The principle of non-maleficence can conflict with the principle of autonomy. If the patient is aware of the potential benefits, the risks and the possible consequences of atrial fibrillation detection, the diagnostic can be done on the user's risk. However, the smartwatches analyzed do not provide any information comparable to traditional medical education. The patient is not well-informed. For informed consent, a more detailed explanation, which allows the user to ask questions and which checks whether the user has understood the explanation, is required. As this is difficult to achieve without human contact, education should be carried out by an experienced doctor.

It is also important to clarify what medical data is stored, who owns the data and how the data is used. By using consumer products in health care, private companies receive sensitive health data that can also be misused for other purposes. Data protection laws must ensure that medical data is not misused and that only the necessary data is collected. If the data will be used for purposes other than health care, the user should give their consent and be informed. The laws shouldn't be over regulatory and thereby hinder research and innovation (22). In addition, private companies must handle data responsibly and be aware of their social responsibility. When the data is evaluated in the context of good scientific practice, both users and society would benefit. To promote innovation and quality, there should be competition and diversity in the market. Small innovative competitors should have access to the infrastructure needed to research big data.

For ensuring safety and liability, the companies must take responsibility for the provision of diagnostic tools. It must be clear who is liable for the accuracy of the algorithm.

Screening for atrial fibrillation with consumer products leads to inequalities in healthcare. Since smartwatches are currently not offered by health insurers, people who cannot afford a smartwatch do not benefit from the advantages. To avoid differences in healthcare access, wearables must also be made available to people with low income. The resulting public expenditure can only be justified with proof of benefit and cost-effectiveness.

The prevalence of atrial fibrillation is highest in the older population (1). It is therefore particularly important that older people are informed and instructed about the application and the correct use of smartwatches for the detection of atrial fibrillation.

The under representation of certain ethnic groups in the training data sets for the algorithms and in study cohorts poses a risk for disparities in access to health care. Due to the variance of algorithmic accuracy depending on the population group, representative studies are required which prove the same accuracy for different ethnic groups (33).

If the ethical principles discussed are applied, smartwatches could offer a great opportunity for the detection of atrial fibrillation and the prevention of adverse events such as stroke. Doctors should select patients who have been shown to benefit from smartwatch-based atrial fibrillation detection. Medical education and an introduction to the correct use of the diagnostic tool, done by a doctor, could lead to informed consent and could guarantee the participation of older people. The patient's autonomy could be increased by proactively participating in monitoring his health. This could also lead to an improved doctor-patient relationship, which could increase compliance.

In summary, it can be said that the use of smartwatches for screening for atrial fibrillation creates ethical challenges. Due to the lack of evidence for an improved outcome, inadequate data protection laws and the lack of sufficient education of the user, the detection of atrial fibrillation with smartwatches cannot be recommended at the moment. If the wearables are equally made available to suitable patients who have been selected and are supervised by a doctor, improved autonomy and quality of life can be assumed.

Further research about the outcome and the number of adverse events due to atrial fibrillation screening is required. In addition, randomized trials are required to assess the impact on the patient's autonomy and the accuracy of the algorithms for different population groups.

## Data Availability Statement

The original contributions generated for this study are included in the article/supplementary materials, further inquiries can be directed to the corresponding author/s.

## Author Contributions

CP conceived and designed the study, acquired and interpreted the data, and wrote the manuscript. FS revised the manuscript critically for important intellectual content. CP and FS approved the manuscript to be published.

## Conflict of Interest

The authors declare that the research was conducted in the absence of any commercial or financial relationships that could be construed as a potential conflict of interest.
